# Hepatic SILAC proteomic data from PANDER transgenic model

**DOI:** 10.1016/j.dib.2016.08.017

**Published:** 2016-08-16

**Authors:** Mark G. Athanason, Stanley M. Stevens, Brant R. Burkhardt

**Affiliations:** University of South Florida, Department of Cell Biology, Microbiology and Molecular Biology, USA

**Keywords:** PANDER, FAM3B, Proteomics, Transgenic, Liver, Lipogenesis

## Abstract

This article contains raw and processed data related to research published in “Quantitative Proteomic Profiling Reveals Hepatic Lipogenesis and Liver X Receptor Activation in the PANDER Transgenic Model” (M.G. Athanason, W.A. Ratliff, D. Chaput, C.B. MarElia, M.N. Kuehl, S.M., Jr. Stevens, B.R. Burkhardt (2016)) [1], and was generated by “spike-in” SILAC-based proteomic analysis of livers obtained from the PANcreatic-Derived factor (PANDER) transgenic mouse (PANTG) under various metabolic conditions [1]. The mass spectrometry output of the PANTG and wild-type B6SJLF mice liver tissue and resulting proteome search from MaxQuant 1.2.2.5 employing the Andromeda search algorithm against the UniprotKB reference database for Mus musculus has been deposited to the ProteomeXchange Consortium (http://www.proteomexchange.org) via the PRIDE partner repository with dataset identifiers PRIDE: PXD004171 and doi:10.6019/PXD004171. Protein ratio values representing PANTG/wild-type obtained by MaxQuant analysis were input into the Perseus processing suite to determine statistical significance using the Significance A outlier test (*p*<0.05). Differentially expressed proteins using this approach were input into Ingenuity Pathway Analysis to determined altered pathways and upstream regulators that were altered in PANTG mice.

**Specifications Table**

**Table:** 

Subject area	*Biology*
More specific subject area	*Liver proteomics*
Type of data	*Tables, figure*
How data was acquired	*Pre-fractionated samples analyzed by LC-MS/MS. Tryptic peptides were separated on a 10* *cm x 75* *cm I.D. reversed phase column packed with 5 μm C18 material with 300* *Å pore size (New Objective) using 125 min gradients of 2–40% ACN in 0.1% formic acid and mass spectrometric analysis performed inline on an Orbitrap XL.*
Data format	*Raw and analyzed*
Experimental factors	*PANcreatic-DERived factor (PANDER) transgenic and wild-type mice were treated under three metabolic conditions: fasted, fed and insulin stimulated. Protein lysate was obtained from liver tissue that was snap-frozen in liquid nitrogen immediately after excision following treatment and euthanasia. Tryptic peptides were generated utilizing the FASP method. Following a desalt procedure, peptides were fractionated by SCX chromatography.*
Experimental features	*Mass spectrometry output was obtained utilizing LC-MS/MS. Survey scans were performed at a resolving power of 60,000 and the top 10 most abundant peaks were selected for subsequent MS/MS analysis.*
Data source location	*Cell Biology, Microbiology and Molecular Biology, CDDI Proteomics Facility. University of South Florida, Tampa, Florida, United States.*
Data accessibility	*Data available within this article and through the ProteomeXchange Consortium via the PRIDE partner repository with dataset identifiers PRIDE:*PXD004171: *Proteomic Evaluation of PANDER TG Mice, Project*doi:10.6019/PXD004171*, Username: reviewer56976@ebi.ac.uk, Password: VtR5rnIR*

**Value of the data**

• This is the first dataset to detail global proteome changes in the liver of the PANDER transgenic mouse model, a disease model of selective hepatic insulin resistance and metabolic disease.
• Biological analysis demonstrated a pleiotropic impact on a variety of functions such as lipid metabolism and numerous cellular functions such as growth, proliferation, death, and signaling.
• This dataset provides a valuable proteomic resource for further metabolic data mining examining PANDER-induced biological functions

## 1 Data

**Fig. 1:**
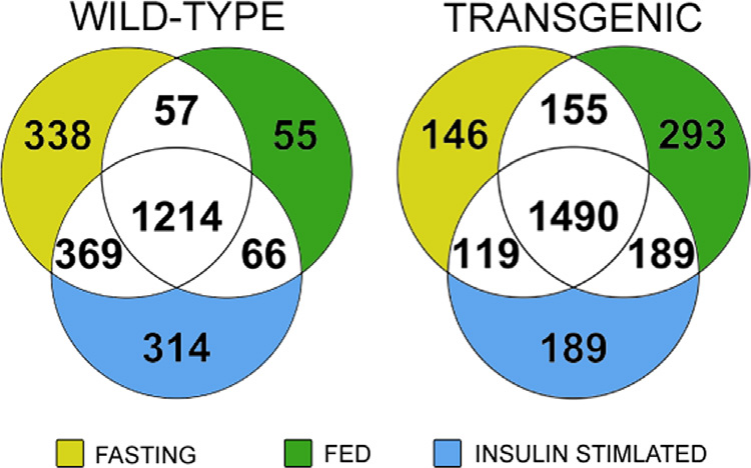
Identification of unique proteins across metabolic states in PANTG. SILAC spike in fractionated protein extracts were analyzed by LC-MS/MS from age- and gender-matched wild-type and PANTG mice (*n*=3 per metabolic condition and genotype). Approximately 3300 proteins were confidently revealed that were shared across genotype and metabolic conditions with overlap indicated in Venn diagram.

SILAC-based proteomic analysis was performed on isolated murine liver tissue following various metabolic treatments obtained from the PANDER transgenic mouse. Of which, 3304 hepatic proteins were identified with analysis comparing PANTG versus wild-type hepatic proteomes within a specific metabolic condition. Of those, there were 228, 239 and 189 differentially expressed proteins in the fasted, fed and insulin-stimulated conditions within the PANTG liver as compared to WT, respectively, as determined by the Significance A outlier test in Perseus (*p*<0.05, *n*=3, combined prior to outlier test). All differentially expressed proteins are listed in Supplementary Tables 1–3 with overall Venn-Diagram of comprehensive results in Fig. 1. A modified ProteinGroups file from MaxQuant showing ratio values for all proteins in each biological replicate along with corresponding RSD values is uploaded in PRIDE under the same accession number.

## 2 Experimental design, materials and methods

### 2.1 PANTG murine model

The proteomic analysis performed in this study utilized the transgenic overexpressing model of PANDER. As previously detailed, this model displays increased circulating levels of PANDER produced from the endocrine pancreas [2].

### 2.2 Metabolic procedures of PANTG

To examine the PANDER-induced hepatic proteome under various metabolic conditions, the PANTG model was exposed to fasting, fed and insulin-stimulated states as detailed in prior manuscript [1].

### 2.3 Liver sample preparation for proteomic analysis

Approximately 40 mg of tissue was excised from a lobe of mouse liver for tissue lysis. Tissue was submerged in cold lysis buffer (100 mM Tris–HCl, 100 mM DTT, 4% SDS and 1xHALT protease inhibitor) and lysed using a Qiagen TissueRupter. Cell lysate was then heated at 95 °C for five minutes followed by brief sonication. The tissue lysate was cleared by centrifugation at 16,000×*g* for 5 min and the supernatant was collected and stored at −80 °C prior to further analysis. The same procedure for protein purification was performed on the “heavy” labeled (^13^C_6_ l-Lysine) liver (Cambridge Isotope Laboratories, Inc).

Protein concentration was quantified using the Pierce 660 nm protein assay kit supplemented with the provided ionic detergent compatibility reagent (IDCR). Equal mass of labeled and unlabeled protein or “light” and “heavy” protein respectively, were combined and digested using the filter-aided sample preparation (FASP) method (Expedeon). Samples were desalted as previously described [1]. Peptides were fractionated by strong cation exchange chromatography as previously described [3].

### 2.4 LC-MS/MS, data processing and pathway analysis

Fractions were separated on a 10 cm×75 μm I.D. reversed phase column packed with 5 μm C18 material with 300 Å pore size (New Objective) using 120 min gradients of 2–40% ACN in 0.1% formic acid. Inline mass spectrometric analysis was performed on a hybrid linear ion trap-Orbitrap mass spectrometer (Orbitrap XL (Thermo Fisher Scientific). Full MS survey scans were performed at a resolving power of 60,000, and the top 10 most abundant peaks were selected for subsequent MS/MS analysis in the linear ion trap. Raw files were processed in MaxQuant 1.2.2.5 employing the Andromeda search algorithm and searched against the UniprotKB reference database for *Mus musculus*, concatenated with reversed protein sequences. A second database of known contaminants provided with the MaxQuant suite was also employed. All fractions for each biological sample were combined for analysis. Constant modification of carbamidomethylation of cysteine and variable modifications of oxidized methionine and acetylated protein N-termini were used. Additionally, Lys-6 for the spike-in internal standard was set as a label in the group-specific parameter section. A false discovery rate of 1% was used for peptides and proteins. A minimum peptide length of 6 amino acids was used. Razor and unique peptides were used for identification and quantification. Protein ratio values were reconstructed using median peptide ratio values across all three biological replicates for each experimental group where the final ratio for each protein was calculated by determining the ratio-of-ratio (PANTG/Internal Standard)/(WT/Internal Standard). Final ratios were input into the Perseus processing suite (Perseus version 1.2.0.13). Statistical analysis was performed using the Significance A outlier test where statistical significance based on magnitude fold-change was established at *p*<0.05. Ratio values and Uniprot Protein identification numbers of differentially expressed proteins were uploaded to Ingenuity Pathway Analysis (IPA). Total number of unique proteins identified within and across metabolic states within the PANTG and WT mice are shown in Fig. 1. Complete listings of all differentially expressed proteins within the PANTG liver as compared to WT identified under the metabolic conditions of fasting, fed, and insulin-stimulated are shown (Supplementary Tables 1–3).In addition, a modified ProteinGroups file from MaxQuant showing protein ratios generated for each biological replicate and with quantifiable relative standard deviation values is also listed (Supplementary Tables 4).

## Appendix A Supplementary material

**Supplementary Table 1.** List of differentially expressed proteins identified during fasting conditions within PANTG liver as compared to WT ranked according to degree of fold change.

**Supplementary Table 2**. List of differentially expressed proteins identified during controlled fed conditions within PANTG liver as compared to WT ranked according to degree of fold change.

**Supplementary Table 3.** List of differentially expressed proteins identified during insulin stimulated conditions within PANTG liver as compared to WT ranked according to degree of fold change.

**Supplementary Table 4. Modified ProteinGroups MaxQuant file.** Modified ProteinGroups file from MaxQuant showing protein ratios generated for each biological replicate with quantifiable related standard deviation values are listed.
