# Supplementary material for: Hepatic SILAC proteomic data from PANDER transgenic model
Source: Data Brief. 2016 Aug 16;9:159–62. doi: 10.1016/j.dib.2016.08.017 (PMC5018088; doi:10.1016/j.dib.2016.08.017)
Supplement: Supplementary file 1 — Supplementary material [file mmc1.doc]

**Conflict of Interest**

The authors do not have any conflict of interest in association with this manuscript.
