# Supplementary material for: Hepatic SILAC proteomic data from PANDER transgenic model
Source: Data Brief. 2016 Aug 16;9:159–62. doi: 10.1016/j.dib.2016.08.017 (PMC5018088; doi:10.1016/j.dib.2016.08.017)
Supplement: Supplementary file 2 — Supplementary Table 1. List of differentially expressed proteins identified during fasting conditions within PANTG liver as compared to WT ranked according to degree of fold change. [file mmc2.doc]

**Supplementary Table 1.**

| **Exp Fold Change** | **ID** | **Symbol** | **Entrez Gene Name** |
| --- | --- | --- | --- |
| 267.090 | E9Q6Z0 | CUL5 | cullin 5 |
| 9.844 | Q8BFW7 | LPP | LIM domain containing preferred translocation partner in lipoma |
| 6.164 | O08663 | METAP2 | methionyl aminopeptidase 2 |
| 5.711 | Q3TKD0 | TNPO1 | transportin 1 |
| 4.723 | P49586 | PCYT1A | phosphate cytidylyltransferase 1, choline, alpha |
| 4.203 | Q9D1P4 | CHORDC1 | cysteine and histidine-rich domain (CHORD) containing 1 |
| 4.004 | P58467 | SETD4 | SET domain containing 4 |
| 3.595 | P58281 | OPA1 | optic atrophy 1 (autosomal dominant) |
| 3.476 | Q9R099 | TBL2 | transducin (beta)-like 2 |
| 3.449 | Q8BYU6 | TOR1AIP2 | torsin A interacting protein 2 |
| 3.403 | B1AU76 | NASP | nuclear autoantigenic sperm protein (histone-binding) |
| 3.284 | D3Z5G7 | Ces1b/Ces1c | carboxylesterase 1C |
| 3.179 | P62960 | YBX1 | Y box binding protein 1 |
| 3.014 | Q01279 | EGFR | epidermal growth factor receptor |
| 2.976 | O35350 | CAPN1 | calpain 1, (mu/I) large subunit |
| 2.835 | Q01339 | APOH | apolipoprotein H (beta-2-glycoprotein I) |
| 2.735 | Q9R0Q6 | ARPC1A | actin related protein 2/3 complex, subunit 1A, 41kDa |
| 2.698 | A3KGU5 | SPTAN1 | spectrin, alpha, non-erythrocytic 1 |
| 2.548 | E9PVM7 | GSTM3 | glutathione S-transferase mu 3 (brain) |
| 2.537 | P01872 | IGHM | immunoglobulin heavy constant mu |
| 2.478 | Q9CRT8 | XPOT | exportin, tRNA |
| 2.467 | P23953 | Ces1b/Ces1c | carboxylesterase 1C |
| 2.395 | P01868 | IGHG1 | immunoglobulin heavy constant gamma 1 (G1m marker) |
| 2.391 | P14901 | HMOX1 | heme oxygenase 1 |
| 2.366 | O35857 | TIMM44 | translocase of inner mitochondrial membrane 44 homolog (yeast) |
| 2.336 | Q9JJU9 | CRYBB3 | crystallin, beta B3 |
| 2.316 | P01029 | C4A/C4B | complement component 4B (Chido blood group) |
| 2.298 | Q8K4H1 | AFMID | arylformamidase |
| 2.229 | Q8BLN5 | LSS | lanosterol synthase (2,3-oxidosqualene-lanosterol cyclase) |
| 2.222 | Q99LM2 | CDK5RAP3 | CDK5 regulatory subunit associated protein 3 |
| 2.175 | Q00898 | SERPINA1 | serpin peptidase inhibitor, clade A (alpha-1 antiproteinase, antitrypsin), member 1 |
| 2.172 | Q9JLC3 | MSRB1 | methionine sulfoxide reductase B1 |
| 2.172 | Q71KU9 | FGL1 | fibrinogen-like 1 |
| 2.133 | P04919 | SLC4A1 | solute carrier family 4 (anion exchanger), member 1 (Diego blood group) |
| 2.085 | Q9R1J0 | NSDHL | NAD(P) dependent steroid dehydrogenase-like |
| 2.057 | K3W4Q8 | BSG | basigin (Ok blood group) |
| 2.042 | Q9R1Q6 | TMEM176B | transmembrane protein 176B |
| 2.001 | Q9D6T0 | NOSIP | nitric oxide synthase interacting protein |
| 1.981 | P06728 | APOA4 | apolipoprotein A-IV |
| 1.978 | Q9DCT8 | Crip2 | cysteine rich protein 2 |
| 1.929 | P15327 | BPGM | 2,3-bisphosphoglycerate mutase |
| 1.925 | P04104 | KRT1 | keratin 1, type II |
| 1.874 | D3YUW7 | CGN | cingulin |
| 1.857 | P08122 | COL4A2 | collagen, type IV, alpha 2 |
| 1.849 | Q8VCR2 | HSD17B13 | hydroxysteroid (17-beta) dehydrogenase 13 |
| 1.844 | P00920 | CA2 | carbonic anhydrase II |
| 1.841 | Q9QXK7 | CPSF3 | cleavage and polyadenylation specific factor 3, 73kDa |
| 1.830 | J3QMN4 | TXNRD2 | thioredoxin reductase 2 |
| 1.816 | P28666 | Mug1/Mug2 | murinoglobulin 1 |
| 1.807 | Q00623 | APOA1 | apolipoprotein A-I |
| 1.805 | Q8CIN4 | PAK2 | p21 protein (Cdc42/Rac)-activated kinase 2 |
| 1.803 | Q8CHW4 | EIF2B5 | eukaryotic translation initiation factor 2B, subunit 5 epsilon, 82kDa |
| 1.803 | Q9CQ01 | RNASET2 | ribonuclease T2 |
| 1.801 | E9PV24 | FGA | fibrinogen alpha chain |
| 1.748 | Q99K23 | UFSP2 | UFM1-specific peptidase 2 |
| 1.740 | P97311 | MCM6 | minichromosome maintenance complex component 6 |
| 1.729 | Q6P1D5 | SEZ6L | seizure related 6 homolog (mouse)-like |
| 1.728 | P13634 | CA1 | carbonic anhydrase I |
| 1.712 | Q8K0E8 | FGB | fibrinogen beta chain |
| 1.692 | P07759 | SERPINA3 | serpin peptidase inhibitor, clade A (alpha-1 antiproteinase, antitrypsin), member 3 |
| 1.676 | Q8K310 | MATR3 | matrin 3 |
| 1.657 | Q9DBE0 | CSAD | cysteine sulfinic acid decarboxylase |
| 1.647 | Q6P2B1 | TNPO3 | transportin 3 |
| 1.640 | Q6ZQ58 | LARP1 | La ribonucleoprotein domain family, member 1 |
| 1.639 | E9Q509 | PKLR | pyruvate kinase, liver and RBC |
| 1.622 | P01837 | IGKC | immunoglobulin kappa constant |
| 1.614 | Q9CQC6 | BZW1 | basic leucine zipper and W2 domains 1 |
| 1.601 | Q8K1J6 | TRNT1 | tRNA nucleotidyl transferase, CCA-adding, 1 |
| 1.594 | Q9JJ28 | FLII | flightless I actin binding protein |
| 1.592 | P60670 | NPLOC4 | NPL4 homolog, ubiquitin recognition factor |
| 1.591 | Q921M3 | SF3B3 | splicing factor 3b, subunit 3, 130kDa |
| 1.586 | Q9DBM2 | EHHADH | enoyl-CoA, hydratase/3-hydroxyacyl CoA dehydrogenase |
| 1.577 | Q5SWU9 | ACACA | acetyl-CoA carboxylase alpha |
| 1.565 | P01867 | Ighg2b | immunoglobulin heavy constant gamma 2B |
| 1.562 | P20918 | PLG | plasminogen |
| 1.561 | Q8BJL9 | UGT2B7 | UDP glucuronosyltransferase 2 family, polypeptide B7 |
| 1.556 | Q9D379 | EPHX1 | epoxide hydrolase 1, microsomal (xenobiotic) |
| 1.551 | Q91VW5 | GOLGA4 | golgin A4 |
| 1.550 | Q8R480 | NUP85 | nucleoporin 85kDa |
| 1.548 | P07309 | TTR | transthyretin |
| 1.545 | Q8BL66 | EEA1 | early endosome antigen 1 |
| 1.535 | P61358 | RPL27 | ribosomal protein L27 |
| 1.528 | Q61171 | PRDX2 | peroxiredoxin 2 |
| 1.522 | A2A977 | CYP4A11 | cytochrome P450, family 4, subfamily A, polypeptide 11 |
| 1.518 | Q99J99 | MPST | mercaptopyruvate sulfurtransferase |
| 1.515 | Q9CXI3 | MOXD1 | monooxygenase, DBH-like 1 |
| 1.508 | Q99PV0 | PRPF8 | pre-mRNA processing factor 8 |
| 1.505 | P26883 | FKBP1A | FK506 binding protein 1A, 12kDa |
| 1.503 | E9QN99 | ABHD14B | abhydrolase domain containing 14B |
| 1.503 | P29699 | AHSG | alpha-2-HS-glycoprotein |
| 1.499 | D3Z3S1 | PREB | prolactin regulatory element binding |
| 1.492 | P43883 | PLIN2 | perilipin 2 |
| 1.489 | P23591 | TSTA3 | tissue specific transplantation antigen P35B |
| 1.486 | E9QKL6 | IFI16 | interferon, gamma-inducible protein 16 |
| 1.486 | Q8K0U4 | HSPA12A | heat shock 70kDa protein 12A |
| 1.485 | Q64435 | UGT1A6 | UDP glucuronosyltransferase 1 family, polypeptide A6 |
| 1.484 | D3YTY9 | KNG1 | kininogen 1 |
| 1.480 | Q8C1A5 | THOP1 | thimet oligopeptidase 1 |
| 1.478 | Q91X72 | HPX | hemopexin |
| 1.473 | E9QA63 | Macf1 | microtubule-actin crosslinking factor 1 |
| 1.470 | P09528 | FTH1 | ferritin, heavy polypeptide 1 |
| 1.469 | Q9CQW9 | IFITM3 | interferon induced transmembrane protein 3 |
| 1.469 | Q9DCY0 | Keg1 | kidney expressed gene 1 |
| 1.467 | Q5SUH7 | CLINT1 | clathrin interactor 1 |
| 1.465 | Q8VCM7 | FGG | fibrinogen gamma chain |
| 1.463 | Q91XL1 | LRG1 | leucine-rich alpha-2-glycoprotein 1 |
| 1.458 | Q9D0E1 | HNRNPM | heterogeneous nuclear ribonucleoprotein M |
| 1.455 | P19096 | FASN | fatty acid synthase |
| 1.446 | P24457 | Cyp2d9 | cytochrome P450, family 2, subfamily d, polypeptide 9 |
| 1.437 | Q61838 | Pzp | pregnancy zone protein |
| 1.430 | Q9EP72 | EMC7 | ER membrane protein complex subunit 7 |
| 1.426 | A2ALV1 | SH3GL2 | SH3-domain GRB2-like 2 |
| 1.425 | Q8BXA1 | GOLIM4 | golgi integral membrane protein 4 |
| 1.423 | P48193 | EPB41 | erythrocyte membrane protein band 4.1 |
| 1.422 | Q9Z1D1 | EIF3G | eukaryotic translation initiation factor 3, subunit G |
| 1.422 | P46935 | Nedd4 | neural precursor cell expressed, developmentally down-regulated 4 |
| 1.422 | P07724 | ALB | albumin |
| 1.422 | Q810B6 | ANKFY1 | ankyrin repeat and FYVE domain containing 1 |
| 1.421 | Q8VBV7 | COPS8 | COP9 signalosome subunit 8 |
| 1.418 | Q5SW19 | CLUH | clustered mitochondria (cluA/CLU1) homolog |
| 1.417 | Q9QXG4 | ACSS2 | acyl-CoA synthetase short-chain family member 2 |
| 1.410 | E9PZJ8 | ASCC3 | activating signal cointegrator 1 complex subunit 3 |
| 1.407 | E0CYY1 | CIAPIN1 | cytokine induced apoptosis inhibitor 1 |
| 1.407 | Q9D6S7 | MRRF | mitochondrial ribosome recycling factor |
| 1.405 | Q3UZ39 | LRRFIP1 | leucine rich repeat (in FLII) interacting protein 1 |
| 1.399 | Q64505 | CYP7A1 | cytochrome P450, family 7, subfamily A, polypeptide 1 |
| 1.396 | P28665 | Mug1/Mug2 | murinoglobulin 1 |
| 1.395 | Q9CY64 | BLVRA | biliverdin reductase A |
| -1.356 | Q9D1M0 | SEC13 | SEC13 homolog, nuclear pore and COPII coat complex component |
| -1.359 | Q8VCC1 | HPGD | hydroxyprostaglandin dehydrogenase 15-(NAD) |
| -1.361 | P17879 | Hspa1b | heat shock protein 1B |
| -1.367 | P26645 | Marcks | myristoylated alanine rich protein kinase C substrate |
| -1.373 | Q61735 | CD47 | CD47 molecule |
| -1.374 | Q9JHJ0 | TMOD3 | tropomodulin 3 (ubiquitous) |
| -1.383 | Q8BK30 | NDUFV3 | NADH dehydrogenase (ubiquinone) flavoprotein 3, 10kDa |
| -1.383 | Q9ERR7 | SEP15 | 15 kDa selenoprotein |
| -1.384 | Q8BH59 | SLC25A12 | solute carrier family 25 (aspartate/glutamate carrier), member 12 |
| -1.384 | P50516 | ATP6V1A | ATPase, H+ transporting, lysosomal 70kDa, V1 subunit A |
| -1.384 | P70671 | IRF3 | interferon regulatory factor 3 |
| -1.386 | Q9D967 | MDP1 | magnesium-dependent phosphatase 1 |
| -1.394 | Q3UID0 | SMARCC2 | SWI/SNF related, matrix associated, actin dependent regulator of chromatin, subfamily c, member 2 |
| -1.396 | P24456 | Cyp2d9 (includes others) | cytochrome P450, family 2, subfamily d, polypeptide 9 |
| -1.405 | Q8VC97 | UPB1 | ureidopropionase, beta |
| -1.408 | Q9D517 | AGPAT3 | 1-acylglycerol-3-phosphate O-acyltransferase 3 |
| -1.411 | Q9WUM3 | CORO1B | coronin, actin binding protein, 1B |
| -1.415 | Q60710 | SAMHD1 | SAM domain and HD domain 1 |
| -1.416 | E9Q557 | DSP | desmoplakin |
| -1.416 | P61750 | ARF4 | ADP-ribosylation factor 4 |
| -1.416 | P63321 | RALA | v-ral simian leukemia viral oncogene homolog A (ras related) |
| -1.417 | Q9R112 | SQRDL | sulfide quinone reductase-like (yeast) |
| -1.419 | F6U2C2 | ATXN2 | ataxin 2 |
| -1.422 | A2AN08 | UBR4 | ubiquitin protein ligase E3 component n-recognin 4 |
| -1.422 | P47754 | CAPZA2 | capping protein (actin filament) muscle Z-line, alpha 2 |
| -1.430 | E9Q4G8 | ALCAM | activated leukocyte cell adhesion molecule |
| -1.433 | E9QAS4 | CHD4 | chromodomain helicase DNA binding protein 4 |
| -1.442 | E9Q8N1 | TTN | titin |
| -1.452 | Q99KJ8 | DCTN2 | dynactin 2 (p50) |
| -1.453 | F8WJK8 | ST13 | suppression of tumorigenicity 13 (colon carcinoma) (Hsp70 interacting protein) |
| -1.455 | Q9JJU8 | SH3BGRL | SH3 domain binding glutamate-rich protein like |
| -1.457 | E9PXX7 | TXNDC5 | thioredoxin domain containing 5 (endoplasmic reticulum) |
| -1.462 | Q2TPA8 | Hsdl2 | hydroxysteroid dehydrogenase like 2 |
| -1.469 | Q3TH01 | HLA-A | major histocompatibility complex, class I, A |
| -1.471 | Q61879 | MYH10 | myosin, heavy chain 10, non-muscle |
| -1.487 | Q61990 | PCBP2 | poly(rC) binding protein 2 |
| -1.488 | Q9R257 | HEBP1 | heme binding protein 1 |
| -1.489 | Q3U0V1 | KHSRP | KH-type splicing regulatory protein |
| -1.494 | P50518 | ATP6V1E1 | ATPase, H+ transporting, lysosomal 31kDa, V1 subunit E1 |
| -1.495 | Q9JLJ5 | ELOVL1 | ELOVL fatty acid elongase 1 |
| -1.495 | Q920A5 | SCPEP1 | serine carboxypeptidase 1 |
| -1.512 | P08752 | GNAI2 | guanine nucleotide binding protein (G protein), alpha inhibiting activity polypeptide 2 |
| -1.524 | Q7TMF3 | NDUFA12 | NADH dehydrogenase (ubiquinone) 1 alpha subcomplex, 12 |
| -1.537 | Q9D1Q6 | ERP44 | endoplasmic reticulum protein 44 |
| -1.549 | P28659 | Celf1 | CUGBP, Elav-like family member 1 |
| -1.550 | Q8VHE0 | SEC63 | SEC63 homolog, protein translocation regulator |
| -1.556 | Q8VCH6 | DHCR24 | 24-dehydrocholesterol reductase |
| -1.574 | Q924Z4 | CERS2 | ceramide synthase 2 |
| -1.584 | P11930 | NUDT19 | nudix (nucleoside diphosphate linked moiety X)-type motif 19 |
| -1.589 | Q07456 | AMBP | alpha-1-microglobulin/bikunin precursor |
| -1.596 | P97855 | G3BP1 | GTPase activating protein (SH3 domain) binding protein 1 |
| -1.623 | A6X935 | ITIH4 | inter-alpha-trypsin inhibitor heavy chain family, member 4 |
| -1.631 | Q921L6 | CTTN | cortactin |
| -1.633 | F8VPN4 | AGL | amylo-alpha-1, 6-glucosidase, 4-alpha-glucanotransferase |
| -1.646 | Q3B7Z2 | OSBP | oxysterol binding protein |
| -1.647 | E9Q8K8 | ZC3H4 | zinc finger CCCH-type containing 4 |
| -1.650 | P45376 | AKR1B1 | aldo-keto reductase family 1, member B1 (aldose reductase) |
| -1.651 | P50462 | CSRP3 | cysteine and glycine-rich protein 3 (cardiac LIM protein) |
| -1.657 | E9Q6R7 | UTRN | utrophin |
| -1.668 | P14094 | ATP1B1 | ATPase, Na+/K+ transporting, beta 1 polypeptide |
| -1.679 | Q8VCC2 | Ces1g | carboxylesterase 1G |
| -1.682 | Q8BWM0 | PTGES2 | prostaglandin E synthase 2 |
| -1.690 | P97792 | CXADR | coxsackie virus and adenovirus receptor |
| -1.691 | Q11136 | PEPD | peptidase D |
| -1.694 | P27612 | PLAA | phospholipase A2-activating protein |
| -1.716 | A2AJK8 | TTC1 | tetratricopeptide repeat domain 1 |
| -1.723 | P02802 | Mt1 | metallothionein 1 |
| -1.792 | Q8R0W0 | EPPK1 | epiplakin 1 |
| -1.810 | Q8R1S9 | SLC38A4 | solute carrier family 38, member 4 |
| -1.826 | H3BJ51 | RETSAT | retinol saturase (all-trans-retinol 13,14-reductase) |
| -1.866 | Q9R092 | HSD17B6 | hydroxysteroid (17-beta) dehydrogenase 6 |
| -1.933 | Q9JI75 | NQO2 | NAD(P)H dehydrogenase, quinone 2 |
| -1.977 | Q8JZK9 | HMGCS1 | 3-hydroxy-3-methylglutaryl-CoA synthase 1 (soluble) |
| -2.006 | Q9EP89 | LACTB | lactamase, beta |
| -2.050 | Q9JHG7 | PIK3CG | phosphatidylinositol-4,5-bisphosphate 3-kinase, catalytic subunit gamma |
| -2.098 | Q9JKB1 | UCHL3 | ubiquitin carboxyl-terminal esterase L3 (ubiquitin thiolesterase) |
| -2.127 | Q9D5T0 | ATAD1 | ATPase family, AAA domain containing 1 |
| -2.134 | Q5XG73 | ACBD5 | acyl-CoA binding domain containing 5 |
| -2.163 | P16546 | SPTAN1 | spectrin, alpha, non-erythrocytic 1 |
| -2.362 | Q9D8Y0 | EFHD2 | EF-hand domain family, member D2 |
| -2.385 | Q9Z329 | ITPR2 | inositol 1,4,5-trisphosphate receptor, type 2 |
| -2.391 | Q9CQH7 | BTF3L4 | basic transcription factor 3-like 4 |
| -2.481 | Q9JLI6 | SCLY | selenocysteine lyase |
| -2.623 | P14115 | RPL27A | ribosomal protein L27a |
| -2.649 | Q9CWL8 | CTNNBL1 | catenin, beta like 1 |
| -2.838 | P35278 | RAB5C | RAB5C, member RAS oncogene family |
| -2.888 | Q9QYC0 | ADD1 | adducin 1 (alpha) |
| -2.897 | Q99MR6 | SRRT | serrate, RNA effector molecule |
| -3.083 | P02463 | COL4A1 | collagen, type IV, alpha 1 |
| -3.154 | Q8R1J1 | TM6SF2 | transmembrane 6 superfamily member 2 |
| -3.171 | Q8CCJ3 | UFL1 | UFM1-specific ligase 1 |
| -3.252 | P61089 | UBE2N | ubiquitin-conjugating enzyme E2N |
| -3.757 | Q9Z2G9 | HTATIP2 | HIV-1 Tat interactive protein 2, 30kDa |
| -3.758 | D3YVR4 | MESDC2 | mesoderm development candidate 2 |
| -3.898 | Q3THK7 | GMPS | guanine monophosphate synthase |
| -4.233 | Q9QYI4 | DNAJB12 | DnaJ (Hsp40) homolog, subfamily B, member 12 |
| -4.307 | O35226 | PSMD4 | proteasome 26S subunit, non-ATPase 4 |
| -4.407 | Q8BH78 | RTN4 | reticulon 4 |
| -4.492 | Q99J77 | NANS | N-acetylneuraminic acid synthase |
| -4.706 | P60229 | EIF3E | eukaryotic translation initiation factor 3, subunit E |
| -4.966 | Q9EQF5 | DPYS | dihydropyrimidinase |
| -5.054 | E9PXN7 | UGT1A7 (includes others) | UDP glucuronosyltransferase 1 family, polypeptide A10 |
| -5.710 | Q91WC0 | SETD3 | SET domain containing 3 |
| -6.314 | B1AZ15 | COBLL1 | cordon-bleu WH2 repeat protein-like 1 |
| -6.517 | P97370 | ATP1B3 | ATPase, Na+/K+ transporting, beta 3 polypeptide |
| -7.087 | P10922 | H1F0 | H1 histone family, member 0 |
| -7.152 | Q9CZW5 | TOMM70A | translocase of outer mitochondrial membrane 70 homolog A (S. cerevisiae) |
| -7.326 | P48024 | Eif1 | eukaryotic translation initiation factor 1 |
| -9.811 | Q91XV3 | BASP1 | brain abundant, membrane attached signal protein 1 |
| -12.682 | Q80W54 | ZMPSTE24 | zinc metallopeptidase STE24 |
| -19.627 | Q8R016 | BLMH | bleomycin hydrolase |
