# Supplementary material for: Hepatic SILAC proteomic data from PANDER transgenic model
Source: Data Brief. 2016 Aug 16;9:159–62. doi: 10.1016/j.dib.2016.08.017 (PMC5018088; doi:10.1016/j.dib.2016.08.017)
Supplement: Supplementary file 3 — Supplementary Table 2. List of differentially expressed proteins identified during controlled fed conditions within PANTG liver as compared to WT ranked according to degree of fold change. [file mmc3.doc]

**Supplementary Table 2.**

| **Exp Fold Change** | **ID** | **Symbol** | **Entrez Gene Name** |
| --- | --- | --- | --- |
| 28.351 | Q8R1S9 | SLC38A4 | solute carrier family 38, member 4 |
| 10.609 | P62960 | YBX1 | Y box binding protein 1 |
| 9.943 | Q61655 | DDX19A | DEAD (Asp-Glu-Ala-Asp) box polypeptide 19A |
| 7.023 | P58044 | IDI1 | isopentenyl-diphosphate delta isomerase 1 |
| 6.515 | Q9ESX5 | DKC1 | dyskeratosis congenita 1, dyskerin |
| 5.497 | Q8BH78 | RTN4 | reticulon 4 |
| 5.266 | Q3UJB0 | SF3B2 | splicing factor 3b, subunit 2, 145kDa |
| 4.886 | Q9JKF1 | IQGAP1 | IQ motif containing GTPase activating protein 1 |
| 4.169 | Q11136 | PEPD | peptidase D |
| 3.783 | Q9Z2G9 | HTATIP2 | HIV-1 Tat interactive protein 2, 30kDa |
| 3.415 | Q14DH7 | ACSS3 | acyl-CoA synthetase short-chain family member 3 |
| 3.376 | Q8VHE0 | SEC63 | SEC63 homolog, protein translocation regulator |
| 3.082 | Q9DCT8 | Crip2 | cysteine rich protein 2 |
| 2.822 | Q9CW03 | SMC3 | structural maintenance of chromosomes 3 |
| 2.629 | Q9CZW5 | TOMM70A | translocase of outer mitochondrial membrane 70 homolog A (S. cerevisiae) |
| 2.457 | Q9CY64 | BLVRA | biliverdin reductase A |
| 2.267 | Q8R0V5 | IDO2 | indoleamine 2,3-dioxygenase 2 |
| 2.200 | Q3TKD0 | TNPO1 | transportin 1 |
| 2.186 | Q99J77 | NANS | N-acetylneuraminic acid synthase |
| 2.170 | Q7TPV4 | MYBBP1A | MYB binding protein (P160) 1a |
| 2.158 | P43883 | PLIN2 | perilipin 2 |
| 2.156 | Q9CQE8 | C14orf166 | chromosome 14 open reading frame 166 |
| 2.143 | O08529 | CAPN2 | calpain 2, (m/II) large subunit |
| 2.061 | Q9CQ80 | VPS25 | vacuolar protein sorting 25 homolog (S. cerevisiae) |
| 2.030 | D3YXZ7 | SLC35A3 | solute carrier family 35 (UDP-N-acetylglucosamine (UDP-GlcNAc) transporter), member A3 |
| 1.995 | Q8VCH0 | Acaa1b | acetyl-Coenzyme A acyltransferase 1B |
| 1.969 | Q5FW60 | Mup1 | major urinary protein 1 |
| 1.943 | Q9JIK5 | DDX21 | DEAD (Asp-Glu-Ala-Asp) box helicase 21 |
| 1.914 | Q921S7 | MRPL37 | mitochondrial ribosomal protein L37 |
| 1.897 | Q8BK64 | AHSA1 | AHA1, activator of heat shock 90kDa protein ATPase homolog 1 (yeast) |
| 1.896 | Q9JJU8 | SH3BGRL | SH3 domain binding glutamate-rich protein like |
| 1.853 | Q8VCI0 | PLBD1 | phospholipase B domain containing 1 |
| 1.845 | B7ZDD7 | CRAT | carnitine O-acetyltransferase |
| 1.827 | Q3UZZ6 | Sult1d1 | sulfotransferase family 1D, member 1 |
| 1.824 | Q9JLV1 | BAG3 | BCL2-associated athanogene 3 |
| 1.773 | Q711T7 | NADSYN1 | NAD synthetase 1 |
| 1.766 | Q99JI6 | RAP1B | RAP1B, member of RAS oncogene family |
| 1.765 | Q8VCC2 | Ces1g | carboxylesterase 1G |
| 1.762 | Q8R0H9 | GGA1 | golgi-associated, gamma adaptin ear containing, ARF binding protein 1 |
| 1.741 | Q8R0J8 | IDNK | idnK, gluconokinase homolog (E. coli) |
| 1.720 | Q91WG0 | Ces2c | carboxylesterase 2C |
| 1.687 | Q3TP97 | TARS2 | threonyl-tRNA synthetase 2, mitochondrial (putative) |
| 1.685 | Q5SQB0 | NPM1 | nucleophosmin (nucleolar phosphoprotein B23, numatrin) |
| 1.684 | E9PZJ8 | ASCC3 | activating signal cointegrator 1 complex subunit 3 |
| 1.680 | Q61635 | Ifi47 | interferon gamma inducible protein 47 |
| 1.648 | F6RWR5 | BC021614 | cDNA sequence BC021614 |
| 1.621 | P15208 | INSR | insulin receptor |
| 1.614 | Q8CEB6 | AKR1E2 | aldo-keto reductase family 1, member E2 |
| 1.612 | D3YYD5 | VPS29 | VPS29 retromer complex component |
| 1.610 | G5E8X1 | C16orf13 | chromosome 16 open reading frame 13 |
| 1.603 | E9Q8N1 | TTN | titin |
| 1.599 | E9PVM7 | GSTM3 | glutathione S-transferase mu 3 (brain) |
| 1.579 | P42703 | LIFR | leukemia inhibitory factor receptor alpha |
| 1.578 | Q8C8T8 | TSR2 | TSR2, 20S rRNA accumulation, homolog (S. cerevisiae) |
| 1.564 | Q8JZK9 | HMGCS1 | 3-hydroxy-3-methylglutaryl-CoA synthase 1 (soluble) |
| 1.554 | Q60737 | CSNK2A1 | casein kinase 2, alpha 1 polypeptide |
| 1.554 | Q9D967 | MDP1 | magnesium-dependent phosphatase 1 |
| 1.549 | Q8CHW4 | EIF2B5 | eukaryotic translation initiation factor 2B, subunit 5 epsilon, 82kDa |
| 1.546 | Q8BGC4 | ZADH2 | zinc binding alcohol dehydrogenase domain containing 2 |
| 1.530 | P19096 | FASN | fatty acid synthase |
| 1.528 | P54823 | DDX6 | DEAD (Asp-Glu-Ala-Asp) box helicase 6 |
| 1.525 | G5E8M7 | Gstm6 | glutathione S-transferase, mu 6 |
| 1.518 | Q3V117 | ACLY | ATP citrate lyase |
| 1.515 | Q8BK30 | NDUFV3 | NADH dehydrogenase (ubiquinone) flavoprotein 3, 10kDa |
| 1.487 | Q8CFB8 | PARP3 | poly (ADP-ribose) polymerase family, member 3 |
| 1.478 | F8WIT2 | ANXA6 | annexin A6 |
| 1.477 | O08738 | CASP6 | caspase 6, apoptosis-related cysteine peptidase |
| 1.471 | O88325 | NAGLU | N-acetylglucosaminidase, alpha |
| 1.466 | Q5HZI1 | MTUS1 | microtubule associated tumor suppressor 1 |
| 1.457 | P63073 | EIF4E | eukaryotic translation initiation factor 4E |
| 1.457 | Q9D023 | MPC2 | mitochondrial pyruvate carrier 2 |
| 1.447 | E9QAA5 | SMPD4 | sphingomyelin phosphodiesterase 4, neutral membrane (neutral sphingomyelinase-3) |
| 1.431 | Q9JI78 | NGLY1 | N-glycanase 1 |
| 1.429 | B1AU76 | NASP | nuclear autoantigenic sperm protein (histone-binding) |
| 1.426 | Q9EQF5 | DPYS | dihydropyrimidinase |
| 1.409 | P24472 | Gsta4 | glutathione S-transferase, alpha 4 |
| 1.407 | Q91WS4 | BHMT2 | betaine--homocysteine S-methyltransferase 2 |
| 1.402 | E9Q7G0 | NUMA1 | nuclear mitotic apparatus protein 1 |
| 1.399 | F6YBC9 | CSNK1A1 | casein kinase 1, alpha 1 |
| 1.387 | Q8C165 | PM20D1 | peptidase M20 domain containing 1 |
| 1.382 | Q9R099 | TBL2 | transducin (beta)-like 2 |
| 1.378 | Q99KR3 | LACTB2 | lactamase, beta 2 |
| 1.377 | P28650 | ADSSL1 | adenylosuccinate synthase like 1 |
| 1.377 | P01887 | B2M | beta-2-microglobulin |
| 1.374 | Q9Z1P6 | NDUFA7 | NADH dehydrogenase (ubiquinone) 1 alpha subcomplex, 7, 14.5kDa |
| 1.373 | Q8VE38 | OXNAD1 | oxidoreductase NAD-binding domain containing 1 |
| 1.369 | Q08857 | CD36 | CD36 molecule (thrombospondin receptor) |
| 1.367 | Q9Z0M5 | LIPA | lipase A, lysosomal acid, cholesterol esterase |
| 1.365 | F8WHU9 | ZPR1 | ZPR1 zinc finger |
| 1.360 | P70677 | CASP3 | caspase 3, apoptosis-related cysteine peptidase |
| 1.359 | Q8BJW6 | EIF2A | eukaryotic translation initiation factor 2A, 65kDa |
| 1.357 | Q8VCZ9 | PRODH2 | proline dehydrogenase (oxidase) 2 |
| 1.357 | P06801 | ME1 | malic enzyme 1, NADP(+)-dependent, cytosolic |
| 1.355 | O88736 | HSD17B7 | hydroxysteroid (17-beta) dehydrogenase 7 |
| 1.352 | Q6ZQI3 | MLEC | malectin |
| 1.350 | Q501J6 | DDX17 | DEAD (Asp-Glu-Ala-Asp) box helicase 17 |
| 1.341 | Q9QXZ6 | Slco1a1 | solute carrier organic anion transporter family, member 1a1 |
| 1.334 | B1AUN3 | EIF2B3 | eukaryotic translation initiation factor 2B, subunit 3 gamma, 58kDa |
| 1.334 | P62342 | SELT | selenoprotein T |
| 1.334 | P97494 | GCLC | glutamate-cysteine ligase, catalytic subunit |
| 1.333 | Q9D6S7 | MRRF | mitochondrial ribosome recycling factor |
| 1.332 | Q8R1S0 | COQ6 | coenzyme Q6 monooxygenase |
| 1.329 | Q64514 | TPP2 | tripeptidyl peptidase II |
| 1.329 | O35350 | CAPN1 | calpain 1, (mu/I) large subunit |
| 1.328 | Q78IK2 | USMG5 | up-regulated during skeletal muscle growth 5 homolog (mouse) |
| 1.322 | Q3UIR3 | DTX3L | deltex 3 like, E3 ubiquitin ligase |
| 1.321 | Q99L20 | Gstt3 | glutathione S-transferase, theta 3 |
| 1.319 | Q9DCM2 | GSTK1 | glutathione S-transferase kappa 1 |
| 1.313 | F6VK94 | TOR1B | torsin family 1, member B (torsin B) |
| 1.311 | Q07797 | LGALS3BP | lectin, galactoside-binding, soluble, 3 binding protein |
| 1.310 | Q9ER72 | CARS | cysteinyl-tRNA synthetase |
| 1.309 | Q8C1B7 | SEPT11 | septin 11 |
| 1.308 | Q9CQ54 | NDUFC2 | NADH dehydrogenase (ubiquinone) 1, subcomplex unknown, 2, 14.5kDa |
| 1.307 | P52792 | GCK | glucokinase (hexokinase 4) |
| 1.301 | Q8R4H7 | NAGS | N-acetylglutamate synthase |
| 1.301 | Q8BXA1 | GOLIM4 | golgi integral membrane protein 4 |
| -1.479 | P11276 | FN1 | fibronectin 1 |
| -1.480 | P26638 | SARS | seryl-tRNA synthetase |
| -1.480 | P28658 | ATXN10 | ataxin 10 |
| -1.487 | Q64511 | TOP2B | topoisomerase (DNA) II beta |
| -1.488 | P42669 | PURA | purine-rich element binding protein A |
| -1.489 | P12815 | PDCD6 | programmed cell death 6 |
| -1.491 | Q9WV54 | ASAH1 | N-acylsphingosine amidohydrolase (acid ceramidase) 1 |
| -1.491 | Q8CI94 | PYGB | phosphorylase, glycogen; brain |
| -1.492 | Q99MN1 | KARS | lysyl-tRNA synthetase |
| -1.501 | Q9WVK4 | EHD1 | EH-domain containing 1 |
| -1.505 | P06683 | C9 | complement component 9 |
| -1.506 | Q99KV1 | DNAJB11 | DnaJ (Hsp40) homolog, subfamily B, member 11 |
| -1.507 | P06728 | APOA4 | apolipoprotein A-IV |
| -1.515 | E9Q555 | RNF213 | ring finger protein 213 |
| -1.517 | D3YZJ1 | SQSTM1 | sequestosome 1 |
| -1.525 | D3Z0R5 | GNPDA1 | glucosamine-6-phosphate deaminase 1 |
| -1.525 | Q9DBB9 | CPN2 | carboxypeptidase N, polypeptide 2 |
| -1.527 | E9PWQ3 | COL6A3 | collagen, type VI, alpha 3 |
| -1.529 | Q9EP69 | SACM1L | SAC1 suppressor of actin mutations 1-like (yeast) |
| -1.547 | P56380 | NUDT2 | nudix (nucleoside diphosphate linked moiety X)-type motif 2 |
| -1.550 | P83940 | TCEB1 | transcription elongation factor B (SIII), polypeptide 1 (15kDa, elongin C) |
| -1.551 | Q8R0J7 | VPS37B | vacuolar protein sorting 37 homolog B (S. cerevisiae) |
| -1.559 | Q62165 | DAG1 | dystroglycan 1 (dystrophin-associated glycoprotein 1) |
| -1.560 | Q8BM72 | HSPA13 | heat shock protein 70kDa family, member 13 |
| -1.568 | Q3UNV4 | Cyp2j13 | cytochrome P450, family 2, subfamily j, polypeptide 13 |
| -1.574 | D6RGA3 | LPCAT3 | lysophosphatidylcholine acyltransferase 3 |
| -1.577 | E9PV24 | FGA | fibrinogen alpha chain |
| -1.581 | Q63886 | UGT1A1 | UDP glucuronosyltransferase 1 family, polypeptide A1 |
| -1.589 | Q9JKX3 | TFR2 | transferrin receptor 2 |
| -1.592 | Q9ESP1 | SDF2L1 | stromal cell-derived factor 2-like 1 |
| -1.600 | Q91XL1 | LRG1 | leucine-rich alpha-2-glycoprotein 1 |
| -1.609 | Q8C872 | TFRC | transferrin receptor |
| -1.618 | P58389 | PPP2R4 | protein phosphatase 2A activator, regulatory subunit 4 |
| -1.618 | Q99K30 | EPS8L2 | EPS8-like 2 |
| -1.626 | P08032 | SPTA1 | spectrin, alpha, erythrocytic 1 |
| -1.646 | P01029 | C4A/C4B | complement component 4B (Chido blood group) |
| -1.648 | Q62313 | Tgoln1 | trans-golgi network protein |
| -1.660 | Q91W64 | Cyp2c70 | cytochrome P450, family 2, subfamily c, polypeptide 70 |
| -1.663 | Q8CCJ3 | UFL1 | UFM1-specific ligase 1 |
| -1.669 | Q61830 | MRC1 | mannose receptor, C type 1 |
| -1.670 | P01027 | C3 | complement component 3 |
| -1.674 | O54984 | ASNA1 | arsA arsenite transporter, ATP-binding, homolog 1 (bacterial) |
| -1.695 | P25233 | NDN | necdin, melanoma antigen (MAGE) family member |
| -1.699 | P15508 | SPTB | spectrin, beta, erythrocytic |
| -1.725 | Q8JZR2 | CRK | v-crk avian sarcoma virus CT10 oncogene homolog |
| -1.726 | P04919 | SLC4A1 | solute carrier family 4 (anion exchanger), member 1 (Diego blood group) |
| -1.735 | P00920 | CA2 | carbonic anhydrase II |
| -1.789 | Q9R257 | HEBP1 | heme binding protein 1 |
| -1.802 | P70194 | CLEC4F | C-type lectin domain family 4, member F |
| -1.805 | Q9CQJ6 | DENR | density-regulated protein |
| -1.806 | Q91WT8 | RBM47 | RNA binding motif protein 47 |
| -1.810 | Q8R0X7 | SGPL1 | sphingosine-1-phosphate lyase 1 |
| -1.831 | Q9R1C7 | PRPF40A | PRP40 pre-mRNA processing factor 40 homolog A |
| -1.833 | Q9QXK7 | CPSF3 | cleavage and polyadenylation specific factor 3, 73kDa |
| -1.836 | Q9CPX6 | ATG3 | autophagy related 3 |
| -1.849 | E9Q179 | GRSF1 | G-rich RNA sequence binding factor 1 |
| -1.855 | Q9D1M7 | FKBP11 | FK506 binding protein 11, 19 kDa |
| -1.856 | G5E8J2 | ANK1 | ankyrin 1, erythrocytic |
| -1.858 | P43275 | Hist1h1a | histone cluster 1, H1a |
| -1.859 | E9QA15 | Cald1 | caldesmon 1 |
| -1.870 | Q8CBM2 | ASPH | aspartate beta-hydroxylase |
| -1.903 | D3Z2Y7 | GPX3 | glutathione peroxidase 3 |
| -1.903 | Q99JF5 | MVD | mevalonate (diphospho) decarboxylase |
| -1.911 | Q9JLC3 | MSRB1 | methionine sulfoxide reductase B1 |
| -1.913 | E9Q4K7 | KIF13B | kinesin family member 13B |
| -1.924 | P48193 | EPB41 | erythrocyte membrane protein band 4.1 |
| -1.928 | Q9DAS9 | GNG12 | guanine nucleotide binding protein (G protein), gamma 12 |
| -1.930 | Q9D8B6 | FAM210B | family with sequence similarity 210, member B |
| -1.940 | Q00896 | SERPINA1 | serpin peptidase inhibitor, clade A (alpha-1 antiproteinase, antitrypsin), member 1 |
| -1.951 | Q64282 | IFIT1B | interferon-induced protein with tetratricopeptide repeats 1B |
| -1.953 | Q8R1Q9 | RBKS | ribokinase |
| -1.971 | E9Q6Z0 | CUL5 | cullin 5 |
| -1.972 | Q80VP1 | EPN1 | epsin 1 |
| -2.000 | E9Q616 | AHNAK | AHNAK nucleoprotein |
| -2.002 | P09813 | APOA2 | apolipoprotein A-II |
| -2.004 | Q6IQY5 | CEP70 | centrosomal protein 70kDa |
| -2.010 | Q8VCC1 | HPGD | hydroxyprostaglandin dehydrogenase 15-(NAD) |
| -2.067 | Q91YR7 | PRPF6 | pre-mRNA processing factor 6 |
| -2.097 | G3UZX6 | SUMO3 | small ubiquitin-like modifier 3 |
| -2.136 | A3KGB4 | TBC1D8B | TBC1 domain family, member 8B (with GRAM domain) |
| -2.143 | Q91X72 | HPX | hemopexin |
| -2.143 | Q80Y55 | BSDC1 | BSD domain containing 1 |
| -2.155 | B1AZ15 | COBLL1 | cordon-bleu WH2 repeat protein-like 1 |
| -2.156 | Q8K0Z7 | TACO1 | translational activator of mitochondrially encoded cytochrome c oxidase I |
| -2.348 | P23249 | MOV10 | Mov10 RISC complex RNA helicase |
| -2.380 | A2A998 | C8A | complement component 8, alpha polypeptide |
| -2.428 | Q9CSU0 | RPRD1B | regulation of nuclear pre-mRNA domain containing 1B |
| -2.432 | Q7TQD2 | TPPP | tubulin polymerization promoting protein |
| -2.484 | P58467 | SETD4 | SET domain containing 4 |
| -2.494 | Q8BGS1 | EPB41L5 | erythrocyte membrane protein band 4.1 like 5 |
| -2.515 | O35226 | PSMD4 | proteasome 26S subunit, non-ATPase 4 |
| -2.696 | E9Q415 | FCGR2B | Fc fragment of IgG, low affinity IIb, receptor (CD32) |
| -2.756 | Q8K1Z0 | COQ9 | coenzyme Q9 |
| -2.813 | P10107 | ANXA1 | annexin A1 |
| -2.862 | Q64505 | CYP7A1 | cytochrome P450, family 7, subfamily A, polypeptide 1 |
| -2.994 | Q05920 | PC | pyruvate carboxylase |
| -3.063 | Q01339 | APOH | apolipoprotein H (beta-2-glycoprotein I) |
| -3.083 | Q61646 | HP | haptoglobin |
| -3.094 | Q8BM55 | TMEM214 | transmembrane protein 214 |
| -3.204 | A6X935 | ITIH4 | inter-alpha-trypsin inhibitor heavy chain family, member 4 |
| -3.296 | Q9CY02 | Ahsp | alpha hemoglobin stabilizing protein |
| -3.451 | P31001 | DES | desmin |
| -3.566 | P48024 | Eif1 | eukaryotic translation initiation factor 1 |
| -3.699 | Q3TUE1 | FUBP1 | far upstream element (FUSE) binding protein 1 |
| -3.729 | E9Q852 | MLLT4 | myeloid/lymphoid or mixed-lineage leukemia; translocated to, 4 |
| -4.531 | Q8R1Q8 | DYNC1LI1 | dynein, cytoplasmic 1, light intermediate chain 1 |
| -4.541 | P11031 | SUB1 | SUB1 homolog, transcriptional regulator |
| -6.484 | Q8CI51 | PDLIM5 | PDZ and LIM domain 5 |
| -6.690 | Q9DBE0 | CSAD | cysteine sulfinic acid decarboxylase |
| -7.503 | Q3UFF7 | LYPLAL1 | lysophospholipase-like 1 |
| -11.102 | Q925P2 | CEACAM1 | carcinoembryonic antigen-related cell adhesion molecule 1 (biliary glycoprotein) |
| -25.054 | P00688 | AMY2B | amylase, alpha 2B (pancreatic) |
