# Supplementary material for: Hepatic SILAC proteomic data from PANDER transgenic model
Source: Data Brief. 2016 Aug 16;9:159–62. doi: 10.1016/j.dib.2016.08.017 (PMC5018088; doi:10.1016/j.dib.2016.08.017)
Supplement: Supplementary file 4 — Supplementary Table 3. List of differentially expressed proteins identified during insulin stimulated conditions within PANTG liver as compared to WT ranked according to degree of fold change. [file mmc4.doc]

**Supplementary Table 3.**

| **Exp Fold Change** | **ID** | **Symbol** | **Entrez Gene Name** | |
| --- | --- | --- | --- | --- |
| 21.746 | Q8BG73 | SH3BGRL2 | SH3 domain binding glutamate-rich protein like 2 | |
| 18.470 | Q8CI94 | PYGB | phosphorylase, glycogen; brain | |
| 15.400 | Q8BFW7 | LPP | LIM domain containing preferred translocation partner in lipoma | |
| 12.723 | P70227 | ITPR3 | inositol 1,4,5-trisphosphate receptor, type 3 | |
| 10.300 | P09813 | APOA2 | apolipoprotein A-II | |
| 9.967 | P47963 | RPL13 | ribosomal protein L13 | |
| 9.156 | P43883 | PLIN2 | perilipin 2 |  |
| 6.153 | Q8R1S9 | SLC38A4 | solute carrier family 38, member 4 | |
| 5.500 | P13516 | SCD | stearoyl-CoA desaturase (delta-9-desaturase) | |
| 5.322 | Q8VCH0 | Acaa1b | acetyl-Coenzyme A acyltransferase 1B | |
| 5.077 | Q64511 | TOP2B | topoisomerase (DNA) II beta | |
| 4.938 | Q91YS7 | MAP2K2 | mitogen-activated protein kinase kinase 2 | |
| 4.832 | P06801 | ME1 | malic enzyme 1, NADP(+)-dependent, cytosolic | |
| 3.609 | J3QML2 | ABCC3 | ATP-binding cassette, sub-family C (CFTR/MRP), member 3 | |
| 3.145 | P62960 | YBX1 | Y box binding protein 1 | |
| 3.072 | Q64458 | CYP2C8 | cytochrome P450, family 2, subfamily C, polypeptide 8 | |
| 2.917 | Q99KR3 | LACTB2 | lactamase, beta 2 | |
| 2.897 | Q8BJL9 | UGT2B7 | UDP glucuronosyltransferase 2 family, polypeptide B7 | |
| 2.894 | H3BL34 | Ces1e | carboxylesterase 1E | |
| 2.856 | P47740 | ALDH3A2 | aldehyde dehydrogenase 3 family, member A2 | |
| 2.834 | Q91X77 | Cyp2c54 (includes others) | cytochrome P450, family 2, subfamily c, polypeptide 54 | |
| 2.765 | Q924Y0 | BBOX1 | butyrobetaine (gamma), 2-oxoglutarate dioxygenase (gamma-butyrobetaine hydroxylase) 1 | |
| 2.747 | Q8VCC2 | Ces1g | carboxylesterase 1G | |
| 2.698 | O54754 | AOX1 | aldehyde oxidase 1 | |
| 2.632 | Q8VHE0 | SEC63 | SEC63 homolog, protein translocation regulator | |
| 2.624 | P26369 | U2AF2 | U2 small nuclear RNA auxiliary factor 2 | |
| 2.616 | Q9ESX5 | DKC1 | dyskeratosis congenita 1, dyskerin | |
| 2.609 | Q9DBM2 | EHHADH | enoyl-CoA, hydratase/3-hydroxyacyl CoA dehydrogenase | |
| 2.607 | Q05D44 | EIF5B | eukaryotic translation initiation factor 5B | |
| 2.561 | G3XA17 | EIF4G2 | eukaryotic translation initiation factor 4 gamma, 2 | |
| 2.510 | Q64459 | CYP3A5 | cytochrome P450, family 3, subfamily A, polypeptide 5 | |
| 2.489 | P28666 | Mug1/Mug2 | murinoglobulin 1 | |
| 2.450 | P24472 | Gsta4 | glutathione S-transferase, alpha 4 | |
| 2.447 | D3Z0Z6 | ELOVL5 | ELOVL fatty acid elongase 5 | |
| 2.407 | P19096 | FASN | fatty acid synthase | |
| 2.403 | Q91X75 | CYP2A6 (includes others) | cytochrome P450, family 2, subfamily A, polypeptide 6 | |
| 2.387 | Q3UP75 | UGT3A1 | UDP glycosyltransferase 3 family, polypeptide A1 | |
| 2.362 | Q9Z211 | PEX11A | peroxisomal biogenesis factor 11 alpha | |
| 2.354 | E9PZJ8 | ASCC3 | activating signal cointegrator 1 complex subunit 3 | |
| 2.336 | H7BX26 | CEP170 | centrosomal protein 170kDa | |
| 2.333 | P16015 | CA3 | carbonic anhydrase III | |
| 2.318 | Q91WG0 | Ces2c | carboxylesterase 2C | |
| 2.286 | Q9D6Y9 | GBE1 | glucan (1,4-alpha-), branching enzyme 1 | |
| 2.245 | Q8BFZ9 | ERLIN2 | ER lipid raft associated 2 | |
| 2.204 | P40936 | INMT | indolethylamine N-methyltransferase | |
| 2.192 | Q62417 | SORBS1 | sorbin and SH3 domain containing 1 | |
| 2.184 | Q6XVG2 | Cyp2c54 (includes others) | cytochrome P450, family 2, subfamily c, polypeptide 54 | |
| 2.172 | Q8BK48 | Ces2e | carboxylesterase 2E | |
| 2.164 | Q8BYU6 | TOR1AIP2 | torsin A interacting protein 2 | |
| 2.157 | J3KMG3 | MIA3 | melanoma inhibitory activity family, member 3 | |
| 2.146 | Q8R480 | NUP85 | nucleoporin 85kDa | |
| 2.141 | P10649 | GSTM5 | glutathione S-transferase mu 5 | |
| 2.126 | Q9DBE0 | CSAD | cysteine sulfinic acid decarboxylase | |
| 2.117 | A2ATU0 | DHTKD1 | dehydrogenase E1 and transketolase domain containing 1 | |
| 2.035 | Q8VCT4 | CES1 | carboxylesterase 1 | |
| 2.029 | Q3V117 | ACLY | ATP citrate lyase | |
| 2.027 | P16045 | LGALS1 | lectin, galactoside-binding, soluble, 1 | |
| 2.016 | P19639 | Gstm3 | glutathione S-transferase, mu 3 | |
| 1.997 | E9Q509 | PKLR | pyruvate kinase, liver and RBC | |
| 1.931 | P35492 | HAL | histidine ammonia-lyase | |
| 1.929 | Q61133 | GSTT2/2B | glutathione S-transferase theta 2 ( | |
| 1.925 | D3YVR4 | MESDC2 | mesoderm development candidate 2 | |
| 1.923 | Q62048 | PEA15 | phosphoprotein enriched in astrocytes 15 | |
| 1.921 | D3Z3E6 | TMEM56 | transmembrane protein 56 | |
| 1.917 | Q9WV68 | DECR2 | 2,4-dienoyl CoA reductase 2, peroxisomal | |
| 1.890 | P55050 | FABP2 | fatty acid binding protein 2, intestinal | |
| 1.888 | Q9WV98 | TIMM9 | translocase of inner mitochondrial membrane 9 homolog (yeast) | |
| 1.884 | Q80WJ7 | MTDH | metadherin | |
| 1.864 | P13634 | CA1 | carbonic anhydrase I | |
| 1.856 | P54728 | RAD23B | RAD23 homolog B, nucleotide excision repair protein | |
| 1.852 | Q921H8 | ACAA1 | acetyl-CoA acyltransferase 1 | |
| 1.839 | Q7TSH2 | PHKB | phosphorylase kinase, beta | |
| 1.829 | Q62264 | THRSP | thyroid hormone responsive | |
| 1.810 | Q99L20 | Gstt3 | glutathione S-transferase, theta 3 | |
| 1.805 | Q91X70 | C6 | complement component 6 | |
| 1.797 | Q91VF2 | HNMT | histamine N-methyltransferase | |
| 1.792 | O88696 | CLPP | caseinolytic mitochondrial matrix peptidase proteolytic subunit | |
| 1.792 | Q9CXN7 | Pbld2 | phenazine biosynthesis-like protein domain containing 2 | |
| 1.788 | Q9D379 | EPHX1 | epoxide hydrolase 1, microsomal (xenobiotic) | |
| 1.785 | Q9WUZ9 | ENTPD5 | ectonucleoside triphosphate diphosphohydrolase 5 | |
| 1.783 | G5E8R3 | PC | pyruvate carboxylase | |
| 1.780 | A2AIH8 | PIR | pirin (iron-binding nuclear protein) | |
| 1.769 | Q9CWS0 | DDAH1 | dimethylarginine dimethylaminohydrolase 1 | |
| 1.769 | F8VPN4 | AGL | amylo-alpha-1, 6-glucosidase, 4-alpha-glucanotransferase | |
| 1.758 | Q5FW57 | Gm4952 | predicted gene 4952 | |
| 1.753 | P56654 | Cyp2c54 (includes others) | cytochrome P450, family 2, subfamily c, polypeptide 54 | |
| 1.747 | P70362 | UFD1L | ubiquitin fusion degradation 1 like (yeast) | |
| 1.747 | P12710 | FABP1 | fatty acid binding protein 1, liver | |
| 1.733 | Q61733 | MRPS31 | mitochondrial ribosomal protein S31 | |
| 1.727 | Q91XT4 | SEC16B | SEC16 homolog B, endoplasmic reticulum export factor | |
| 1.726 | Q9CQR4 | ACOT13 | acyl-CoA thioesterase 13 | |
| 1.719 | Q91WU0 | Ces1f | carboxylesterase 1F | |
| 1.718 | Q8QZR3 | Ces2a | carboxylesterase 2A | |
| 1.707 | Q8VIJ6 | SFPQ | splicing factor proline/glutamine-rich | |
| 1.706 | Q3TI14 | MRPS23 | mitochondrial ribosomal protein S23 | |
| 1.683 | Q9DB26 | PHYHD1 | phytanoyl-CoA dioxygenase domain containing 1 | |
| 1.680 | Q9R0H0 | ACOX1 | acyl-CoA oxidase 1, palmitoyl | |
| 1.673 | Q8K4H1 | AFMID | arylformamidase | |
| 1.666 | G5E8M7 | Gstm6 | glutathione S-transferase, mu 6 | |
| 1.660 | P99025 | GCHFR | GTP cyclohydrolase I feedback regulator | |
| 1.651 | P55096 | ABCD3 | ATP-binding cassette, sub-family D (ALD), member 3 | |
| 1.643 | Q8R0V5 | IDO2 | indoleamine 2,3-dioxygenase 2 | |
| 1.642 | O70194 | EIF3D | eukaryotic translation initiation factor 3, subunit D | |
| 1.639 | Q9QUH0 | GLRX | glutaredoxin (thioltransferase) | |
| 1.626 | Q91V76 | C11orf54 | chromosome 11 open reading frame 54 | |
| 1.616 | P16406 | ENPEP | glutamyl aminopeptidase (aminopeptidase A) | |
| 1.597 | G3X982 | Aox3 | aldehyde oxidase 3 | |
| -1.695 | P21981 | TGM2 | transglutaminase 2 | |
| -1.696 | Q923D5 | WBP11 | WW domain binding protein 11 | |
| -1.698 | P97855 | G3BP1 | GTPase activating protein (SH3 domain) binding protein 1 | |
| -1.711 | P46638 | RAB11B | RAB11B, member RAS oncogene family | |
| -1.713 | Q3TML0 | PDIA6 | protein disulfide isomerase family A, member 6 | |
| -1.724 | Q61247 | SERPINF2 | serpin peptidase inhibitor, clade F (alpha-2 antiplasmin, pigment epithelium derived factor), member 2 | |
| -1.727 | Q9CRD0 | OCIAD1 | OCIA domain containing 1 | |
| -1.738 | P09528 | FTH1 | ferritin, heavy polypeptide 1 | |
| -1.739 | Q3TDQ1 | STT3B | STT3B, subunit of the oligosaccharyltransferase complex (catalytic) | |
| -1.761 | E9PYH3 | ETNPPL | ethanolamine-phosphate phospho-lyase | |
| -1.762 | P08113 | HSP90B1 | heat shock protein 90kDa beta (Grp94), member 1 | |
| -1.765 | P51150 | RAB7A | RAB7A, member RAS oncogene family | |
| -1.765 | Q61699 | HSPH1 | heat shock 105kDa/110kDa protein 1 | |
| -1.781 | E9QNH6 | MYO1B | myosin IB |  |
| -1.783 | P57716 | NCSTN | nicastrin |  |
| -1.797 | Q9ESP1 | SDF2L1 | stromal cell-derived factor 2-like 1 | |
| -1.799 | Q9QZ85 | Iigp1 | interferon inducible GTPase 1 | |
| -1.810 | Q99KF1 | TMED9 | transmembrane p24 trafficking protein 9 | |
| -1.813 | P04919 | SLC4A1 | solute carrier family 4 (anion exchanger), member 1 (Diego blood group) | |
| -1.838 | P06683 | C9 | complement component 9 | |
| -1.844 | O55137 | Acot1 | acyl-CoA thioesterase 1 | |
| -1.853 | Q64339 | ISG15 | ISG15 ubiquitin-like modifier | |
| -1.867 | P01867 | Ighg2b | immunoglobulin heavy constant gamma 2B | |
| -1.874 | Q8CHP5 | PYM1 | PYM homolog 1, exon junction complex associated factor | |
| -1.878 | P14901 | HMOX1 | heme oxygenase 1 | |
| -1.884 | P01837 | IGKC | immunoglobulin kappa constant | |
| -1.908 | P58021 | TM9SF2 | transmembrane 9 superfamily member 2 | |
| -1.933 | E9PV24 | FGA | fibrinogen alpha chain | |
| -1.940 | Q8VC97 | UPB1 | ureidopropionase, beta | |
| -1.985 | Q60991 | CYP7B1 | cytochrome P450, family 7, subfamily B, polypeptide 1 | |
| -2.001 | O70400 | PDLIM1 | PDZ and LIM domain 1 | |
| -2.011 | Q9DBG7 | SRPR | signal recognition particle receptor (docking protein) | |
| -2.015 | G3X9T8 | CP | ceruloplasmin (ferroxidase) | |
| -2.015 | Q9D8B6 | FAM210B | family with sequence similarity 210, member B | |
| -2.016 | P50462 | CSRP3 | cysteine and glycine-rich protein 3 (cardiac LIM protein) | |
| -2.020 | P53994 | RAB2A | RAB2A, member RAS oncogene family | |
| -2.033 | P01027 | C3 | complement component 3 | |
| -2.034 | O35226 | PSMD4 | proteasome 26S subunit, non-ATPase 4 | |
| -2.047 | P70441 | SLC9A3R1 | solute carrier family 9, subfamily A (NHE3, cation proton antiporter 3), member 3 regulator 1 | |
| -2.058 | Q80ZP8 | MANF | mesencephalic astrocyte-derived neurotrophic factor | |
| -2.063 | Q99NB9 | SF3B1 | splicing factor 3b, subunit 1, 155kDa | |
| -2.066 | Q8K0E8 | FGB | fibrinogen beta chain | |
| -2.131 | P01029 | C4A/C4B | complement component 4B (Chido blood group) | |
| -2.165 | Q8BLN5 | LSS | lanosterol synthase (2,3-oxidosqualene-lanosterol cyclase) | |
| -2.167 | Q9CQJ6 | DENR | density-regulated protein | |
| -2.179 | E9QA15 | Cald1 | caldesmon 1 | |
| -2.185 | Q8BWN8 | ACOT4 | acyl-CoA thioesterase 4 | |
| -2.229 | O35864 | COPS5 | COP9 signalosome subunit 5 | |
| -2.233 | Q9EQ32 | PIK3AP1 | phosphoinositide-3-kinase adaptor protein 1 | |
| -2.246 | H3BKR2 | GNB1 | guanine nucleotide binding protein (G protein), beta polypeptide 1 | |
| -2.253 | Q91V77 | S100A1 | S100 calcium binding protein A1 | |
| -2.275 | B1AQR8 | LGALS9B | lectin, galactoside-binding, soluble, 9B | |
| -2.276 | Q8K0Z7 | TACO1 | translational activator of mitochondrially encoded cytochrome c oxidase I | |
| -2.278 | P62264 | RPS14 | ribosomal protein S14 | |
| -2.330 | Q8K2Q0 | COMMD9 | COMM domain containing 9 | |
| -2.352 | P58281 | OPA1 | optic atrophy 1 (autosomal dominant) | |
| -2.352 | P11276 | FN1 | fibronectin 1 | |
| -2.359 | Q91X72 | HPX | hemopexin | |
| -2.361 | Q61990 | PCBP2 | poly(rC) binding protein 2 | |
| -2.368 | Q8VCM7 | FGG | fibrinogen gamma chain | |
| -2.402 | Q9D7X3 | DUSP3 | dual specificity phosphatase 3 | |
| -2.423 | P17879 | Hspa1b | heat shock protein 1B | |
| -2.487 | A6X935 | ITIH4 | inter-alpha-trypsin inhibitor heavy chain family, member 4 | |
| -2.516 | Q99K30 | EPS8L2 | EPS8-like 2 | |
| -2.520 | P97315 | CSRP1 | cysteine and glycine-rich protein 1 | |
| -2.636 | Q8CGR7 | UPP2 | uridine phosphorylase 2 | |
| -2.825 | E9PUZ8 | C4bp | complement component 4 binding protein | |
| -2.834 | Q9QXK7 | CPSF3 | cleavage and polyadenylation specific factor 3, 73kDa | |
| -2.861 | P32921 | WARS | tryptophanyl-tRNA synthetase | |
| -2.934 | Q9D1J3 | SARNP | SAP domain containing ribonucleoprotein | |
| -2.949 | P01868 | IGHG1 | immunoglobulin heavy constant gamma 1 (G1m marker) | |
| -3.288 | Q923B6 | STEAP4 | STEAP family member 4 | |
| -3.436 | E0CYI9 | GCC2 | GRIP and coiled-coil domain containing 2 | |
| -3.825 | Q01279 | EGFR | epidermal growth factor receptor | |
| -3.944 | P47809 | MAP2K4 | mitogen-activated protein kinase kinase 4 | |
| -4.097 | Q8BJW6 | EIF2A | eukaryotic translation initiation factor 2A, 65kDa | |
| -4.131 | D3Z368 | CAMK1 | calcium/calmodulin-dependent protein kinase I | |
| -5.378 | Q61646 | HP | haptoglobin | |
| -5.563 | P62281 | RPS11 | ribosomal protein S11 | |
| -6.881 | P60229 | EIF3E | eukaryotic translation initiation factor 3, subunit E | |
| -7.557 | O55239 | NNMT | nicotinamide N-methyltransferase | |
| -13.618 | Q9JIK5 | DDX21 | DEAD (Asp-Glu-Ala-Asp) box helicase 21 | |
